# Evaluating Physiological and Hormonal Responses of Two Distinct Rice Genotypes Under High Temperatures

**DOI:** 10.3390/plants14050710

**Published:** 2025-02-26

**Authors:** Xiaoyu Qi, Weicai Jin, Wenhao Zhong, Jiatong Han, Muhammad Afzal, Qiang Yue, Guoping Wang, Mehmood Jan

**Affiliations:** 1 College of Horticulture, South China Agricultural University, Guangzhou 510642, China; qixiaoyu0628@163.com (X.Q.); mafzal@scau.edu.cn (M.A.); gpwang@scau.edu.cn (G.W.); 2 Guangdong Provincial Key Laboratory of Utilization and Conservation of Food and Medicinal Resources in Northern Region, Shaoguan University, Shaoguan 512005, China; yueqiang1997@163.com; 3 College of Agriculture, South China Agriculture University, Guangzhou 510642, China; xdfjinweicai@163.com (W.J.); whwh@stu.scau.edu.cn (W.Z.); hanjiatong@stu.scau.edu.cn (J.H.)

**Keywords:** anthesis, flowering, spikelet fertility, abscisic acid (ABA), hydrogen peroxide, APX

## Abstract

Climate change poses a major threat to rice productivity, particularly due to high-temperature stress during anthesis, which severely impacts the grain yield. Understanding the physiological and biochemical responses of different rice genotypes to high-temperature stress is critical for breeding resilient varieties. In this study, we assessed two contrasting rice genotypes, high-temperature-tolerant-1 (HTR-1) and high-temperature-sensitive (HTS-5), to confirm previously established physiological and hormonal mechanisms associated with high-temperature tolerance. The study evaluated morphological, physiological, and biochemical markers at the anthesis stage under control (29/24 °C) and high-temperature stress (38 °C for six hours) conditions. Our results confirmed that HTR-1 exhibits superior tolerance through better antioxidant enzyme activity, higher anther dehiscence, and lower oxidative damage. The genotype HTS-5 exhibited a substantial rise in hydrogen peroxide (1.9-fold) and malondialdehyde (1.74-fold) levels, accompanied by the reduced activity of antioxidant enzymes. Furthermore, the high transcript level of cytosolic APX (*OsAPX1*, *OsAPX2*), peroxisomal APX (*OsAPX3* and *OsAPX4*), *OsCATA*, and *OsCATB* confirmed high antioxidant activity in HTR-1. Moreover, the GA and IAA levels were reduced in both genotypes, while the ABA concentration was increased significantly in the anthers of HTS-5 as compared to those of HTR-1. This suggests that higher ABA production, along with higher reactive oxygen species (ROS) in the anthers, could lead to sterility in rice under high-temperature scenarios. These findings confirmed HTR-1 as a promising genetic resource for breeding heat-tolerant rice, by validating physiological and biochemical mechanisms of high-temperature resilience. This study also provides practical insights for selecting suitable genotypes to improve rice production under the challenges of climate change.

## 1. Introduction

In the present era, abiotic stress factors pose a substantial challenge to global food production, which must keep pace with the escalating demands of a rapidly growing population [1,2]. Among these factors, high temperatures are a major environmental challenge that adversely impacts all living organisms, including plants [3]. The projections of global climate change suggest that the average global temperature could rise about 1.0 to 3.7 °C by the end of this century compared to the period between 1850 and 1900 [2,4]. Anthropogenic activities are the primary drivers of environmental disruptions with industrial emissions of greenhouse gases, including nitrous oxides, methane, chlorofluorocarbons, and notably CO_2_, significantly elevating atmospheric greenhouse gas concentrations and exacerbating climate variability [5]. In recent years, climate change has intensified the occurrence of extreme weather events, such as heat waves, which pose serious threats to grain crop yields [6,7].

Rice (*Oryza sativa* L.) is a thermophilic crop, with optimal developmental temperatures ranging between 24 °C and 29 °C during the night and day. Temperatures exceeding this range can impose stress on the plant, adversely affecting its growth, development, and reproductive processes [8,9]. To fulfill the increasing demand of the world’s growing population, the rice yield must be enhanced by 40% by 2040 [2]. Rice is particularly sensitive to heat stress during the reproductive phase, especially at anthesis, where high temperatures can impair the pollen viability and anther dehiscence, leading to a reduced grain set and yield losses [10]. Reductions in the yield have been documented in numerous tropical and subtropical regions globally, including China, India, and Pakistan [11,12]. It is estimated that in 2030, a 10% decline in rice yields will occur in South Asian countries due to an increase in the annual temperatures [13]. Thus, it is vital to identify and create new rice varieties with increased heat tolerance for breeding programs to alleviate these negative consequences.

High-temperature stress has been shown to initiate the production of reactive oxygen species (ROS), including hydrogen peroxide (H_2_O_2_), hydroxyl (OH), and superoxide (O_2_.^−^), which inflict considerable damage on plant cellular machinery [2,14,15]. Recent research has emphasized the important role of H_2_O_2_ in stress tolerance in different plant species [16]; however, at higher concentrations, H_2_O_2_ can induce programmed cell death (PCD) [17]. A study on cytoplasmic male sterility in *Oryza sativa* L. has shown that ROS-induced PCD is directly linked to pollen sterility [18].

To mitigate ROS and maintain redox homeostasis, plants have developed a complex antioxidant defense system comprising several vital enzymes, including glutathione (GR), catalase (CAT), peroxidase (POD), ascorbate peroxidase (APX) and superoxide dismutase (SOD) [19]. In rice, the regulation of *APX* genes has been confirmed by previous researchers in response to both biotic and environmental stresses, including high temperatures [14,20]. The over-expression of cytosolic *APX* genes has been revealed to enhance the plant’s ability to scavenge H_2_O_2_, thereby protecting it against salt and cold stress [21]. Catalases are also essential, facilitating the breakdown of H_2_O_2_ from peroxisomes and mitochondria [22]. Additionally, water stress adversely impacts pollen development, and drought-resistant cultivars exhibit higher yields and more effective activities of POD, CAT, and SOD compared to sensitive cultivars [23].

Plants are immobile and particularly susceptible to environmental stressors such as high temperatures, which significantly modulate phytohormone levels and consequently affect development and the yield. In *Arabidopsis*, it has been demonstrated that indole-3-acetic acid (IAA) regulates pollen development and anther dehiscence and also helps in pre-anthesis filament elongation [24]. Furthermore, the exogenous application of IAA has been shown to reverse the sterility induced by high temperatures in *Arabidopsis* and barley [25]. Additionally, gibberellins (GAs) are also critical for anther and pollen production and for the proper development of pollen tubes [26] and also influence the flowering time just before anthesis [27]. Abscisic acid (ABA) is a well-explored phytohormone due to its crucial role in regulating the plant stress response [28]. Variation in ABA levels can lead to male sterility and modulate various physico-biochemical processes during exposure to temperature stress [29]. Additionally, exogenous ABA has been shown to induce pollen sterility at the young microspore stage of anther maturation [30].

Numerous studies have established the role of ROS homeostasis, antioxidant activity, and phytohormonal regulation in high-temperature stress responses, and the direct evaluation of tolerant and sensitive rice genotypes remains valuable for breeding applications. This study aimed to confirm the physiological, biochemical, and hormonal responses of two distinct rice genotypes, HTR-1 and HTS-5, when exposed to high-temperature stress during anthesis. By validating HTR-1’s improved antioxidant defense, lower oxidative damage, and more stable hormonal balance under high-temperature conditions, this study supports its potential as a genetic resource for breeding heat-tolerant rice. Overall, these findings contribute to breeding efforts by identifying a high-temperature-tolerant genotype as a promising candidate for breeding new rice varieties suitable for high temperatures.

## 2. Materials and Methods

### 2.1. Experimental Design and Plant Growth Conditions

Two Indica (*Oryza sativa* L.) genotypes, Zao89-554, high-temperature-tolerant-1 (HTR-1), and Zhan-07, high-temperature-sensitive (HTS-5), were selected for experimentation based on the preliminary experimental results. The plant materials were obtained from the College of Agronomy, South China Agriculture University.

To break seed dormancy, the seeds were maintained at 40 °C for one week. The seeds were then immersed in double-distilled water at a temperature of 30 °C for two to three days, followed by incubation at 37.5 °C for one day. Subsequently, the uniformly germinated seeds were transferred into seedling trays (40 × 30 × 12 cm) containing 16 kg of clay loam soil. After 21 days, the seedlings were transferred to pots (21 × 16 cm) filled with 3 kg of soil. The soil composition comprised 54% silt, 14% clay, and 32% sand. Before planting, the soil was thoroughly mixed with 1 g of single superphosphate, 2 g of ammonium sulfate [(NH_4_)_2_SO_4_], and 1 g of potassium chloride. An additional 2 g of ammonium sulfate was applied 30 to 35 days after planting.

The plants were grown under controlled temperature conditions within a greenhouse, maintained at 29 °C during the day and 24 °C at night, with a consistent relative humidity of 70% throughout the growth cycle. To prevent overcrowding effects, plants were spaced 25–30 cm apart. Additionally, pots were rearranged every two weeks to ensure uniform environmental conditions for plant growth. The water level in each pot was maintained at a constant level at 3 cm throughout the experiment.

### 2.2. High-Temperature Treatment

The plants were transferred from the greenhouse to an automated growth chamber at 8:00 AM on the day of anthesis. The chamber’s temperature was gradually increased from 29 °C to 38 °C by 8:30 AM (increased by 3 °C every 10 min) and then maintained at 38 °C for 6 h, until 2:30 PM, with a relative humidity of 70%. Following the temperature treatment, plants were returned to their initial growth conditions of 29 °C/24 °C during the day/night, respectively. This temperature treatment was repeated for four consecutive days. Since rice plants do not flower simultaneously, separate groups of plants were used for the treatment, ensuring they were spaced 60–70 cm apart during the process.

### 2.3. Anther Collection

Anther samples were collected from 30 to 40 plants exposed to either high-temperature conditions (38 °C) or the ambient temperature (29 °C) from opened flowers. The samples were rapidly frozen in liquid nitrogen and stored at −80 °C until further analysis.

### 2.4. Observations

#### 2.4.1. Panicle Exertion (PE), Peduncle Length (PeL), and Flowering Period (FP)

The panicle exertion and peduncle length were measured with a ruler as previously described by Rang et al. (2011) [31]. The flowering period (FP) was recorded as the number of days required for the main tillers to complete flowering in both genotypes.

#### 2.4.2. Microscopic Observations of Spikelets

Fifteen to twenty spikelets were carefully collected from the main tiller during the flowering period, ensuring minimal disturbance. The spikelets were sampled two hours after stress exposure and immediately placed in Falcon tubes containing a fixative solution composed of 18% double-distilled water, 5% acetic acid, 50% absolute ethanol, and 27% formaldehyde.

#### 2.4.3. Anthers Dehiscence and Undehiscence

The dehisced and undehisced anthers were counted using a stereomicroscope (Leica MZ95, St. Gallen, Switzerland) following the method described by Jagadish et al. (2007) [32]. Anthers exhibiting either an apical or basal pore were classified as dehisced, whereas those lacking such pores were categorized as undehisced. Anther samples were collected 2 h into treatment, and the dehiscence percentage was calculated as the proportion of dehisced anthers relative to the total number observed.

#### 2.4.4. Pollen Germination on Stigmas

After rinsing them with double-distilled water, the spikelets were dissected under a stereomicroscope (Leica MZ95, Switzerland). The stigmas were then incubated in 8 N sodium hydroxide solution at 25 °C for 8–10 h, followed by staining with 0.1% aniline blue in 0.1 M K_2_HPO_4_. The total number of pollen grains and the number of germinated pollen grains on each stigma were recorded, and images were captured using a camera (Nikon DS-Ri1, Tokyo, Japan) attached to the microscope (Nikon AZ 100), following the protocol described by Rang et al. (2011) [31]. Pollen grains were considered germinated if their pollen tube length was at least equal to the diameter of the grain [33].

#### 2.4.5. Spikelet Fertility Percentage

The spikelet fertility (SF) was determined for the main tiller following the method described by Li et al. (2015) [34]. Spikelets that opened between 8:30 and 14:30 during treatment were marked using acrylic markers, with different colors assigned to distinguish each day (red for the 1st day, black for the 2nd day, blue for the 3rd day, and yellow for the 4th day). Fifteen to twenty days after anthesis, the marked spikelets were assessed for their fertility percentage. Their fertility was evaluated by gently pressing each spikelet between the thumb and forefinger to distinguish filled from empty spikelets, with both fully filled and partially filled spikelets classified as fertile. The spikelet fertility was measured over two consecutive years to ensure consistency and reliability.

#### 2.4.6. Pollen Viability

Fluorescein diacetate dye was used to assess the pollen viability before and after treatment. A stock solution was prepared by dissolving 0.02 g/mL fluorescein diacetate in 100 µL of acetone and kept at −20 °C. For the viability assay, 1 µL of the stock solution was diluted with 1 mL of an MOPS buffer, following the protocol described by Heslop-Harrison et al. (1970) [35].

### 2.5. Qualitative and Quantitative Analysis of H_2_O_2_ and O_2_.^−^

The hydrogen peroxide (H_2_O_2_) levels were quantified using the Amplex Red hydrogen peroxide/peroxidase assay kit (Thermo Fisher Scientific, Waltham, MA, USA) according to the protocol of Le et al. (2016) [36], with minor modifications. Frozen anthers were finely ground into a powder using liquid nitrogen and transferred to a pre-cooled 2 mL centrifuge tube. An ice-cold phosphate buffer (100 µL) was added, and the mixture was centrifuged at 13,500 rpm for 5 min at 4 °C. The supernatant was collected for further analysis, and the assay was performed according to the manufacturer’s instructions.

For H_2_O_2_ staining, anthers were incubated in a freshly prepared 3,3′-diaminobenzidine (DAB) solution at 22 °C in the dark, following the protocol outlined by Sun et al. (2013) [37]. The samples were then de-stained using a 4:1 ethanol to chloroform solution, transferred to a glycerol solution, and stored in the dark. Images were captured using a Leica MZ95 microscope (Switzerland). The superoxide anion (O_2_.^−^) levels were determined according to the method described by Munir et al. (2024) [38].

### 2.6. Malondialdehyde (MDA) Determination

Frozen anthers were homogenized in 5% trichloroacetic acid (TCA) and centrifuged at 13,500 rpm for 12 min. The resulting supernatant (2 mL) was then combined with 1 mL of 0.5% thiobarbituric acid prepared in 20% TCA. The reaction mixture was incubated in a water bath at 100 °C for 30 min. After incubation, the samples were rapidly cooled on ice and centrifuged, and the absorbance was measured at 450 nm, 532 nm, and 600 nm, following the method described by Fu et al. (2011) [39].

### 2.7. Antioxidant Assay

The antioxidant activities were measured using a modified protocol. Frozen anthers were ground into a fine powder using liquid nitrogen, homogenized in a phosphate buffer (pH 7.0), and centrifuged at 13,000 rpm for 15 min at 4 °C. The superoxide dismutase (SOD) activity was determined following the method described by Islam et al. (2016) [19]. For the assessment of the ascorbate peroxidase (APX) activity, frozen anthers were separately ground and homogenized in a phosphate buffer (50 mM, pH 7.0) containing 0.1 mM Na_2_EDTA, 1 mM ascorbic acid (AsA), and 0.5% (*w*/*v*) polyvinylpyrrolidone (PVP). The resulting supernatant was transferred to a new tube and stored at −80 °C until further analysis. The APX activity was measured by following the protocol explained by Nakano et al. (1981) [40]. The peroxidase (POD) activity was quantified following the method described by Nakurte et al. (2012) [41], while the catalase (CAT) activity was assessed according to the protocol outlined by Islam et al. (2016) [19].

### 2.8. Determination of Plant Phytohormones

The endogenous levels of indole-3-acetic acid (IAA), abscisic acid (ABA), and gibberellic acid (GA) were quantified using ELISA kits (Rapiobio, San Diego, CA, USA) following the manufacturer’s protocol. Frozen anthers were crushed in liquid nitrogen and extracted with HPLC-grade methanol. The extract was centrifuged at 4000× *g* for 12 min at room temperature. The resulting supernatant was transferred to a fresh tube and vacuum-evaporated to reduce the volume to one-tenth of the original. The residue was dissolved in a 1% acetic acid solution and filtered through a 0.2 µm membrane filter. The samples were further purified using C18 solid-phase extraction (SPE) columns as previously described by Nakurte et al. (2012) [42] before phytohormone quantification.

### 2.9. RNA Extraction, cDNA, and qRT-PCR Analysis

Fresh anthers were homogenized into a fine powder in liquid nitrogen, and the total RNA was extracted using Trizol reagent according to the manufacturer’s protocol. The extracted RNA was reverse-transcribed into complementary DNA (cDNA) using the Prime Script™ RT kit (TaKaRa, Kyoto, Japan). Quantitative real-time PCR (qRT-PCR) was performed using a LightCycler 96 system (Roche, Basel, Switzerland) with SYBR Premix Ex Taq (TaKaRa, Japan). Rice Actin 1 was used as the internal reference gene [16]. The data were analyzed following the method outlined by Livak et al. (2001) [43]. The primers used in this study are listed in Supplementary Table S1.

### 2.10. Statistical Analysis

The experimental data are presented as the means ± the standard error from four replicates for hormonal, antioxidant, and ROS determination. Physiological observations (FP, EPE, PeL) were recorded for 10 replicates. Microscopic observations were noted for the flowers of 8 plants that opened during treatment. Statistical analysis was conducted using SPSS (Version 19.0) with a two-way ANOVA followed by Duncan’s multiple-range test to identify significant differences among the treatment means at a 5% significance level. The gene expression data are reported as the means ± the standard error from three biological replicates, each with two technical replicates.

## 3. Results

### 3.1. Phenotypic Response of Rice Genotypes Under High-Temperature Stress

**Figure 1:**
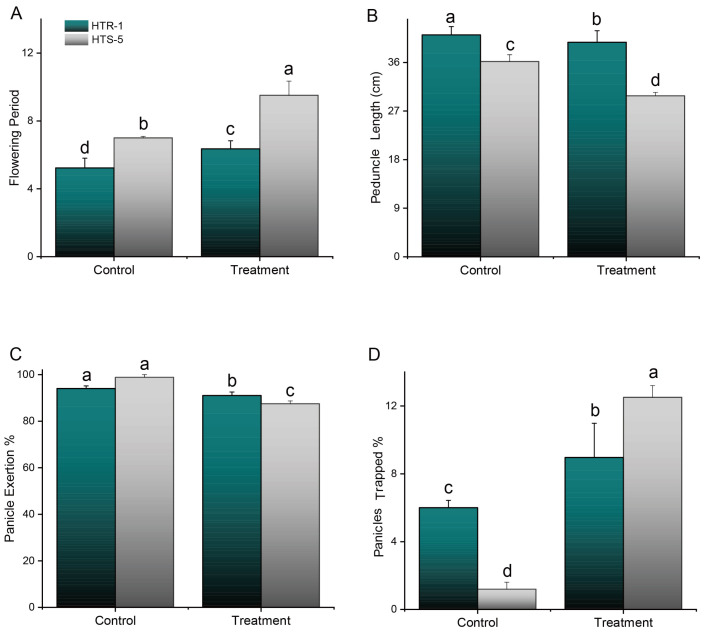
Effect of high temperatures on the (**A**) flowering period (days), (**B**) peduncle length (cm), (**C**) panicle exertion percentage, and (**D**) panicles trapped percentage in two rice genotypes (HTR-1 and HTS-5) subjected to high-temperature stress. Different letters indicate a significant difference between treatments.

To evaluate the impact of high temperatures on the flowering period (FP), peduncle length (PL), panicle exertion (PE), and panicles trapped (PT), both genotypes were subjected to high-temperature stress. The results demonstrated that the PL and PE were significantly reduced by high temperatures in both genotypes compared with the control (Figure 1), while the FP was significantly prolonged (*p* < 0.05) under high temperatures. Under normal conditions, the FP was 5.23 days in HTR-1 and 7 days in HTS-5. However, exposure to high temperatures significantly extended the FP to 6.35 days in HTR-1 and 9.50 days in HTS-5. Additionally, HTR-1 exhibited a higher PL than HTS-5 under both control and high-temperature treatments. Under stress, the PL was significantly reduced from 36.2 cm to 29.8 cm in HTS-5. Due to incomplete panicle exertion under high temperatures, the PE and PT percentages were measured from the flag leaf collar. The percentage of PT increased under high-temperature conditions compared to that of the control. Under normal conditions, 6% of the panicles in HTR-1 and 1.2% in HTS-5 were trapped within the flag leaf collar. However, under high-temperature stress, the PT percentage increased to 8.96 in HTR-1 and 12.5% in HTS-5, highlighting a substantial increase in panicle trapping under high temperatures (Figure 1).

### 3.2. Anther Dehiscence, Pollen Deposition, and Germination Were Negatively Impacted by High Temperatures

**Figure 2:**
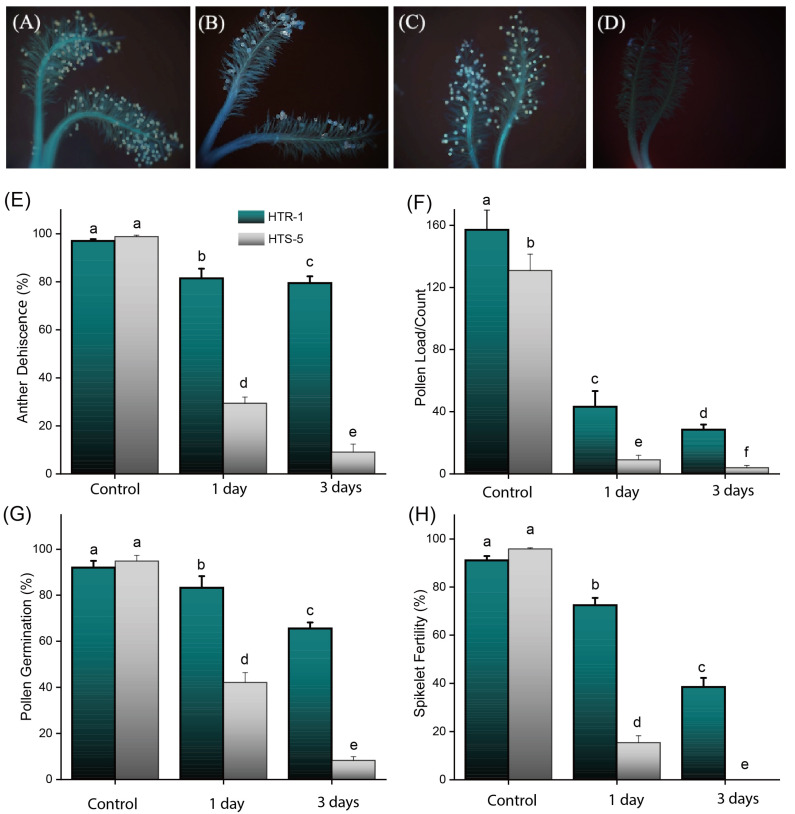
Pictorial illustration of stigmas (**A**–**D**): (**A**) HTR-1 control, (**B**) HTR-1 under high-temperature stress, (**C**) HTS-5 control, (**D**) HTS-5 under high-temperature stress. (**E**) Anther dehiscence Percentage, (**F**) pollen load/count, (**G**) pollen germination Percentage, (**H**) spikelet fertility Percentage. Bars on figures indicate standard error, and values with different letters indicate significant differences at *p* ≤ 0.05 as determined by Duncan’s test.

Anthesis is a crucial and highly temperature-sensitive stage in the rice life cycle, during which damage can lead to significant yield loss. Microscopic observations revealed that anther dehiscence (AD) was severely affected by high-temperature stress, with significant differences observed between the genotypes (Figure 2). HTS-5 exhibited a highly significant difference (*p* < 0.01) in AD compared to HTR-1 under high-temperature stress. Under a controlled environment, no differences in AD were observed between the genotypes. However, under high temperatures, HTR-1 exhibited a moderate decline in AD, reducing to 81.56% and 79.62% after one and three days of high-temperature stress, respectively. In contrast, HTS-5 showed a more drastic reduction in AD, dropping to 29.45% and 9.1% after one and three days of stress (Figure 2E).

High-temperature stress also influenced pollen deposition on the stigmas, with HTR-1 retaining more pollen grains than HTS-5 (Figure 2F). After one day of heat exposure, the number of pollen grains per stigma significantly declined to 43.33 in HTR-1 and 9.2 in HTS-5. After three days of stress, pollen deposition was further reduced to 28.63 and 6 grains per stigma in HTR-1 and HTS-5, respectively. After three days of high-temperature exposure, pollen germination was reduced to 65.62% in HTR-1 and dropped sharply to 8.30% in HTS-5, relative to their respective controls (Figure 2F,G). These findings indicate that HTR-1 exhibited a significantly better retention of AD, pollen deposition, and pollen germination under high-temperature stress compared to HTS-5, suggesting greater resilience to high temperatures.

### 3.3. Impact of High-Temperature Stress on Spikelet Fertility

High-temperature stress significantly reduced the spikelet fertility (SF) percentage in both genotypes (Figure 2H and Figure S1). The SF was 91.26% and 95.91% in HTR-1 and HTS-5 under controlled conditions (29 °C). However, after one day of high-temperature stress (38 °C for six hours), the SF declined significantly to 72.56% in HTR-1 and 15.45% in HTS-5. With prolonged exposure to high temperatures, the SF continued to decline significantly (*p* < 0.05) in both genotypes (Figure S1A). After three days of high-temperature treatment, the SF in HTR-1 was further reduced to 38%, whereas HTS-5 exhibited complete sterility, with no seed fertility observed. These results suggest that HTR-1 demonstrates better tolerance to high-temperature stress, while HTS-5 is highly sensitive, leading to complete sterility under high temperatures.

### 3.4. Determination of Pollen Viability Under High Temperatures

**Figure 3:**
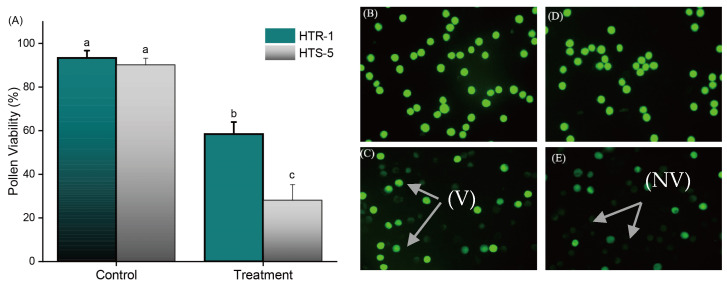
Effect of high temperatures on (**A**) pollen viability percentage (%); pictorial illustration of pollen viability (**B**–**E**): (**B**) HTR-1 control, (**C**) HTR-1 high-temperature treatment, (**D**) HTS-5 control, (**E**) HTS-5 under high-temperature stress. Bars on figure indicate standard error, and values with different letters indicate significant difference at *p* ≤ 0.05, determined by Duncan’s test. V shows viable pollen, while NV shows non-viable.

The pollen metabolic viability was assessed at 5 to 10 min intervals following the initiation of temperature stress (8:30 AM). The results revealed a significant decline in the pollen viability after 35 min of high-temperature exposure in opened spikelets (Figure 3A–E). Under controlled conditions, no significant differences in the pollen viability were observed between both genotypes. However, under high-temperature stress, the pollen viability was reduced by 42.67% in HTR-1 and by 75% in HTS-5, highlighting a greater sensitivity of HTS-5 to high temperatures. High-temperature stress has a profound impact on the crop yield, particularly in the context of climate change, where heat waves are becoming more frequent and intense. Our findings indicate that pollen is highly sensitive to high temperatures, which adversely affect pollen germination on the stigmas, pollen tube growth, and fertilization, ultimately leading to a poor seed set and reduced crop yield.

### 3.5. H_2_O_2_ Quantification, Histochemical Localization, and O_2_.^−^ and MDA Analyses

**Figure 4:**
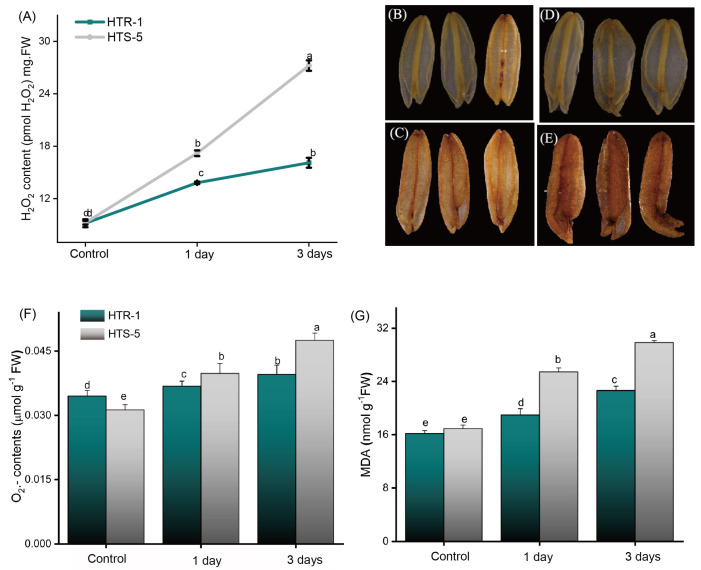
Effect of high-temperature stress on (**A**) H_2_O_2_ contents in rice anthers (%). Pictorial illustration of H_2_O_2_ using DAB (**B**–**E**): (**B**) HTR-1 control, (**C**) HTR-1 high-temperature treatment, (**D**) HTS-5 control, (**E**) HTS-5 high-temperature treatment. (**F**) O_2_.^−^ contents and (**G**) malonaldehyde (MDA) levels. Bars on figures indicate standard error, and values with different letters indicate significant difference at *p* ≤ 0.05, determined by Duncan’s test.

The production of H_2_O_2_ in the anthers was quantified in both genotypes, revealing a 1.9-fold increase in HTS-5 and a 1.5-fold increase in HTR-1 after the first day of high temperatures (Figure 4A–E). After three days of temperature stress, a further significant accumulation of H_2_O_2_ was observed in the anthers of both genotypes (Figure 4A). Additionally, H_2_O_2_ localization in high-temperature-stressed anthers was observed via DAB staining, where HTS-5 anthers exhibited a more intense brown coloration compared to those of HTR-1, indicating higher H_2_O_2_ accumulation (Figure 4B–E). The levels of superoxide anion (O_2_.^−^) also increased more prominently in HTS-5 than in HTR-1, with HTS-5 anthers showing significantly higher O_2_.^−^ accumulation after three days of stress (Figure 4F). Furthermore, oxidative damage was assessed by measuring the malondialdehyde (MDA) levels, an indicator of lipid peroxidation. The MDA levels increased by 1.54-fold and 1.74-fold in HTS-5 after one and three days of high-temperature treatment, respectively (Figure 4G). Collectively, these results demonstrate that high-temperature stress induces significantly greater oxidative damage in HTS-5 compared to HTR-1, suggesting that HTS-5 is more susceptible to temperature-induced oxidative stress.

### 3.6. Antioxidant Enzyme Activities and Their Transcript Expression in Anthers

**Figure 5:**
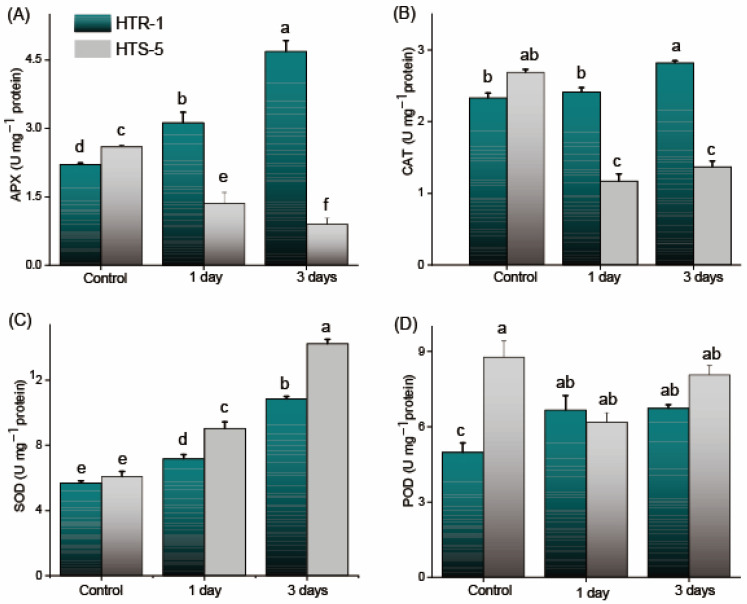
Influence of high temperatures on the (**A**) ascorbate peroxidase (APX), (**B**) catalase (CAT), (**C**) superoxide dismutase (SOD), and (**D**) peroxidase (POD) levels of two rice genotypes (HTR-1 and HTS-5) with n = 4 measurements. Bars on the figures indicate a standard error, and the values with different letters indicate significant differences at *p* ≤ 0.05, determined by Duncan’s test.

Antioxidant enzyme activities play a crucial role in maintaining ROS homeostasis in plants. To assess the impact of high-temperature stress on ROS-scavenging antioxidant enzymes, their activities were measured in the anthers under both control and heat stress conditions (Figure 5). The activities of APX and CAT significantly increased in HTR-1 following one and three days of high-temperature treatment, exhibiting a linear rise in the response to heat stress (Figure 5A,B). In contrast, the APX and CAT activities were markedly inhibited in HTS-5 anthers under high-temperature stress (Figure 5A,B), suggesting a weaker antioxidant defense mechanism in HTS-5 compared to HTR-1. The SOD activity in HTS-5 increased significantly by 1.51-fold and 2.4-fold after one and three days of heat stress, respectively, which was comparatively higher than the increase observed in HTR-1 (Figure 5C). The activity of POD declined in HTS-5 anthers by 29% and 8% after one and three days of treatment, respectively. In contrast, the POD activity in HTR-1 anthers exhibited a significant increase compared to the control conditions, with no substantial variation between the first and third days of heat exposure (Figure 5D). Overall, these results indicate that HTR-1 maintains a more robust antioxidant defense system under high-temperature stress, while HTS-5 exhibits a compromised antioxidant response, making it more susceptible to oxidative damage.

### 3.7. Phytohormone Metabolism in Anthers Under High Temperatures

**Figure 6:**
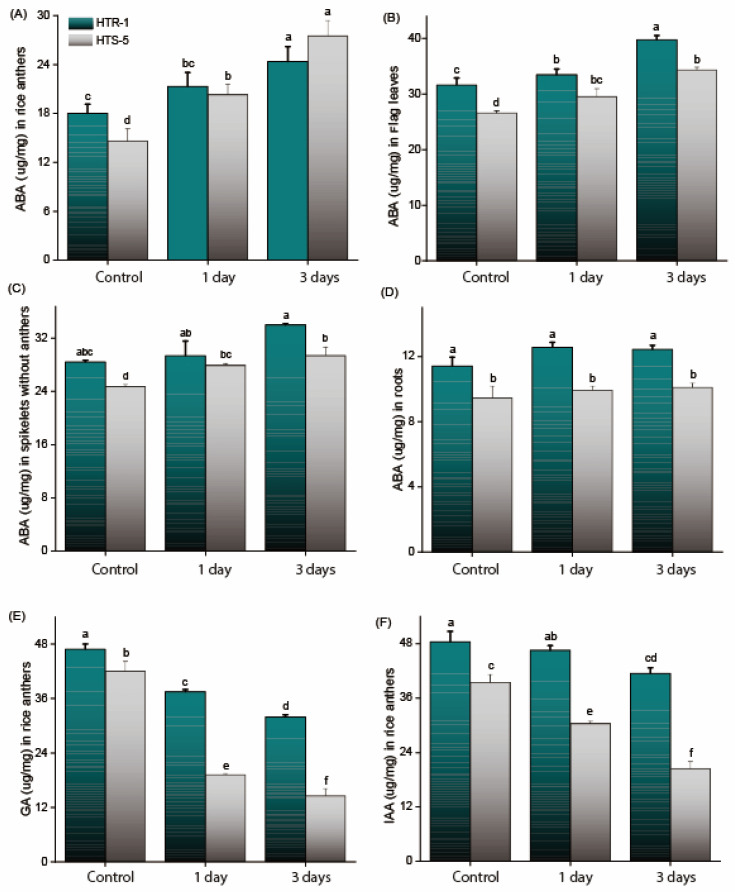
Influence of high temperatures on (**A**) ABA contents in rice anthers, (**B**) ABA contents in flag leaves, (**C**) ABA contents in spikelets, (**D**) ABA contents in roots, (**E**) GA contents in anthers, and (**F**) IAA contents in anthers of two rice genotypes (HTR-1 and HTS-5) with n = 4 measurements. Bars on figures indicate standard error, and values with different letters indicate significant differences at *p* ≤ 0.05, determined by Duncan’s test.

To elucidate the relationship between high-temperature stress and phytohormonal regulation during anthesis, the endogenous levels of ABA, GA, and IAA were measured in the anthers (Figure 6). Under control conditions, HTR-1 exhibited a significantly higher basal level of endogenous ABA compared to HTS-5. However, upon exposure to high-temperature stress, ABA biosynthesis increased in the anthers of both genotypes, with a more pronounced accumulation observed in HTS-5. The ABA levels in HTS-5 anthers increased by 38% and 96% after one and three days of temperature stress, respectively, indicating a strong temperature-induced ABA response in this genotype (Figure 6A). Additionally, the ABA levels were quantified in the flag leaves, spikelets (excluding anthers), and roots under high-temperature conditions (Figure 6B–D). ABA accumulation significantly increased in the flag leaves and spikelets of both genotypes after three days of temperature stress. Conversely, no significant change was observed in the ABA levels within root tissues following high-temperature treatment (Figure 6D).

In contrast to ABA, the GA and IAA levels were significantly reduced in the anthers under high-temperature stress in both genotypes (Figure 6E,F). In HTS-5, the GA levels decreased by 45% and 64.3% after one and three days of temperature exposure, respectively (Figure 6E). Similarly, the IAA content in HTS-5 declined by 26% and 52% after one and three days of temperature stress, respectively, compared to the control conditions (Figure 6F). Altogether, these findings indicate that high-temperature stress disrupts the phytohormonal balance in anthers, characterized by increased ABA accumulation and reduced GA and IAA levels. This hormonal imbalance likely contributes to poor anther dehiscence, impaired pollination, and reduced fertilization, with HTS-5 displaying greater sensitivity to temperature stress than HTR-1.

### 3.8. High Temperatures Impacted the Relative Expression of Antioxidant- and ABA-Related Genes

**Figure 7:**
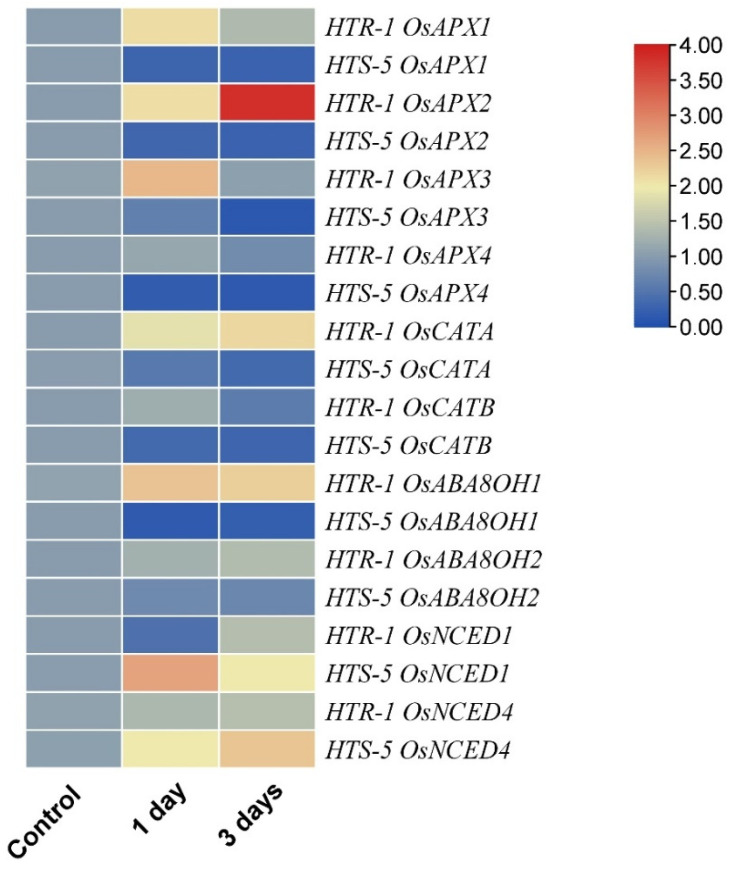
Effect of high temperatures on the relative expression of genes coding for APX and CAT enzymes and ABA metabolism in the anthers of two rice genotypes (HTR-1 and HTS-5).

To elucidate the differential activity patterns of antioxidants in the anthers of different rice genotypes, the transcript levels of *OsCAT* and *OsAPX* were analyzed via qPCR (Figure 7). Under high-temperature stress, *OsCATA* and *OsCATB* were significantly upregulated in HTR-1 compared to HTS-5 (Figure 7). Among the APX genes, *OsAPX*1, *OsAPX*2, *OsAPX*3, and *OsAPX*4 were significantly downregulated in the anthers of HTS-5 under high-temperature conditions (Figure 7). Conversely, in HTR-1, *OsAPX1*, *OsAPX2*, and *OsAPX3* exhibited significant upregulation after one and three days of high-temperature treatment, whereas *OsAPX4* expression was reduced by 0.2-fold after three days of stress. These results suggest that higher expression levels of antioxidant enzyme genes (*OsAPX*, *OsSOD*, *OsPOD*, and *OsCAT*) in HTR-1 contribute to better protection against high-temperature-induced oxidative stress compared to that of HTS-5.

Additionally, the expression of ABA biosynthesis and catabolism genes (*OsNCED1*, *OsNCED4*, *OsABA8OH1*, and *OsABA8OH2*) was quantified to examine the impact of high-temperature stress on ABA metabolism. *OsNCED1* expression was downregulated in HTR-1 after one day of temperature stress, whereas it was upregulated by 1.7-fold in HTS-5. After three days, the *OsNCED1* expression in HTR-1 returned to control levels, while it remained higher in HTS-5 (Figure 7). Similarly, *OsNCED4* expression increased significantly in HTS-5 after one and three days of high-temperature stress, whereas HTR-1 exhibited only a slight increase. In contrast, *OsABA8OH1* and *OsABA8OH2* were upregulated considerably in HTR-1 but downregulated in HTS-5 following one and three days of temperature stress (Figure 7). These findings indicate that high-temperature stress induces distinct changes in ABA metabolism, alongside alterations in IAA and GA levels, contributing to pollen sterility in the temperature-sensitive genotype HTS-5.

## 4. Discussion

Climate change, particularly rising temperatures, poses a significant threat to rice growth and yields, with the flowering stage being especially vulnerable when temperatures exceed the critical threshold of 33  °C. Fluctuations during this stage can lead to infertility and a reduced grain yield [34]. To reduce high-temperature-induced yield losses during flowering, three key strategies can be employed: (1) heat escape, achieved through an early-morning flowering time to avoid peak temperatures [44,45]; (2) temperature avoidance, facilitated by a transpiration-mediated cooling process [46]; and (3) temperature tolerance, where resilient physiological and reproductive processes, such as anther dehiscence, pollination efficiency, pollen germination on the stigmas, fertilization, and a sustained seed-setting ratio, enable the plant to withstand high temperatures [31]. However, to accurately determine the true temperature tolerance in rice, it is critical to ensure that the crop is not merely escaping or avoiding high-temperature stress via the aforementioned heat escape and avoidance strategies. A deeper understanding of intrinsic temperature tolerance mechanisms is essential for developing climate-resilient rice varieties and ensuring sustainable rice production in the face of rising global temperatures.

This study confirms that high temperatures have a negative impact on rice flowering, growth, and morphology. Our findings are consistent with previous studies that show that crops exhibit an extended flowering time under abiotic stress conditions [31,47].

Notably, the flowering period was significantly extended in the high-temperature-sensitive (HTS-5) genotype compared to the high-temperature-resistant (HTR-1) genotype (Figure 1). The prolonged flowering time could be a potential strategy for avoiding high temperatures [48], but in the present study, it did not enhance the seed fertility under stress conditions. Panicle exertion (PE) plays a critical role in grain yield determination under high-temperature stress, as insufficient PE can result in a shortened peduncle length, causing spikelets to become trapped within the leaf sheath [33]. In this study, spikelets enclosed within the flag leaf sheath were completely sterile, probably due to increased internal temperatures and limited airflow [31]. These findings show the importance of optimal panicle exertion for ensuring reproductive success under high-temperature stress and highlight HTS-5’s susceptibility to heat-induced sterility.

Previous studies have established that the reproductive stage in rice is more vulnerable to abiotic stresses than the vegetative stage [49,50]. In this study, high-temperature stress negatively regulated anther dehiscence, leading to a reduced number of pollen grains on the stigmas (Figure 2A). The existing literature suggests that poor anther dehiscence, impaired pollen reception [2,51], the reduced apical or basal pore size of the anthers, and disturbances in pollen swelling [49,52] are critical factors contributing to a decline in pollen deposition and germination, ultimately resulting in severe yield losses in rice [33,53] and sorghum [54].

Our findings align with previous studies, which indicated that high temperatures significantly reduced the pollen viability [50], which reduced the pollen availability and germination efficiency on the stigmas (Figure 2). The primary cause of reduced germinated pollen on the stigmas was pollen retention within the anthers due to high-temperature stress. The results demonstrate a strong correlation between the pollen viability, anther dehiscence, and germinated pollen count on the stigmas and the spikelet fertility, which likely explains the decreased seed set and lower harvest index under temperature stress (Figure 2H). Notably, previous research suggests that at least 10 germinated pollen grains per stigma are essential to maintain high spikelet fertility under high-temperature conditions [31,32], further reinforcing our findings on the impact of temperature stress on rice’s reproductive success.

High-temperature stress disrupts the balance between ROS production and scavenging, leading to oxidative stress. Normally, ROS levels peak during tapetum degeneration and pollen maturation in rice and *Arabidopsis thaliana* [55,56] but are absent in the later stages of anther differentiation [55]. Our results confirmed that high-temperature stress significantly increased H_2_O_2_, MDA, and O_2_.^−^ accumulation, with HTS-5 showing much higher levels than the tolerant genotype (Figure 4). These findings are consistent with previous reports showing increased ROS levels in various crop species under abiotic stress conditions [39,54].

In our study, high-temperature stress significantly affected antioxidant enzyme activities, particularly those of SOD, APX, and CAT (Figure 5). While the APX and CAT activities were strongly upregulated in HTR-1 under high-temperature stress, all enzymes were significantly inhibited in HTS-5, with the exception of SOD. The enhanced APX and CAT activities in HTR-1 suggest their crucial role in scavenging excessive H_2_O_2_, thereby mitigating oxidative stress under temperature stress conditions [5,57]. Additionally, the expression of tomato cytosolic/peroxisomal APX was significantly upregulated in anthers under temperature stress, highlighting its role in preventing or repairing heat-induced oxidative damage as a component of thermotolerance [57]. Further evidence suggests that APX expression and activity in the anthers protect male reproductive organs from oxidative stress through both ROS-dependent and ROS-independent mechanisms, driven by heat shock elements in their promoters [58,59]. These findings reinforce the importance of antioxidant enzyme regulation in maintaining reproductive viability under high-temperature stress.

Plants, being sessile, rely on complex signaling networks to perceive and adapt to adverse environmental conditions, ensuring the successful completion of their life cycle. Among these signaling mechanisms, phytohormones play a crucial role in regulating male reproductive development under stress conditions. In line with previous findings [60], our study shows that high-temperature stress significantly reduced the GA and IAA levels, with a more significant reduction in the sensitive genotype compared to the tolerant genotype (Figure 6). Similarly, cold stress has been reported to reduce bioactive gibberellins (GA_7_ and GA_4_) in developing rice anthers, further emphasizing the temperature sensitivity of GA homeostasis during reproductive development [61]. Gibberellins are essential for anther development and pollen viability [26], and GA deficiency has been linked to abnormal anther growth and male sterility in tomatoes, petunias, rice, and Arabidopsis [62,63]. Likewise, IAA homeostasis in anthers during the flowering stage is critical, as any disruption in its levels may compromise the anther’s defense response against high-temperature stress [64]. A reduction in the endogenous IAA levels can impair pollen embryogenesis and accelerate pollen maturation, leading to developmental defects [65]. Our findings indicate that decreased GA and IAA levels were strongly associated with impaired anther dehiscence and reduced spikelet fertility under high-temperature stress. These results confirm the importance of maintaining GA and IAA homeostasis for reproductive resilience in rice under high-temperature stress.

Abscisic acid (ABA) is a key phytohormone that regulates various physiological processes throughout the plant life cycle, including responses to environmental stress. In this study, high-temperature stress led to increased ABA levels in both genotypes, with a negative correlation between high-temperature tolerance and ABA accumulation (Figure 6A). Previous studies have shown that excessive ABA accumulation in the anthers induces sterility in rice [30], chickpeas, and wheat under cold and drought stress [66]. Similarly, exogenous ABA application repressed the expression of *TaIVR1*, causing sterility in wheat by disrupting sugar metabolism and pollen development in the anthers [67]. High-temperature stress significantly influenced the expression of ABA biosynthetic and catabolic genes during anthesis (Figure 7). To validate the temperature-induced expression pattern of *OsNCED4*, anther samples were collected from both genotypes in the morning (10 AM) and afternoon (2:30 PM) under normal conditions, followed by qPCR analysis. The results revealed the downregulation of *OsNCED4* in both genotypes at 2:30 PM (Figure S1B). However, under high-temperature stress, *OsNCED4* expression was significantly upregulated in both genotypes, with a notably higher increase in HTS-5 (Figure 7). In response to temperature stress, ABA catabolic genes (*OsABA8OH1* and *OsABA8OH2*) were significantly upregulated in HTR-1, suggesting an efficient ABA degradation mechanism, whereas both genes were downregulated in HTS-5, leading to excessive ABA accumulation. Additionally, the ABA levels in flag leaves and spikelets increased after three days of high-temperature stress in both genotypes, suggesting that ABA synthesis primarily occurred in the anthers rather than ABA being transported from other tissues (Figure 6A–D). These findings indicate that the differential regulation of ABA biosynthetic (*OsNCED4*) and catabolic (*OsABA8OH1* and *OsABA8OH2*) genes contributes to the contrasting responses of HTR-1 and HTS-5 under high-temperature stress. In summary, these findings highlight potential differences in ROS scavenging and ABA accumulation between the two genotypes, which may influence reproductive success under high-temperature stress during anthesis. The observed variations in ABA levels and antioxidant enzyme activities suggest that heat tolerance in HTR-1 may be linked to a more balanced hormonal and oxidative stress response. However, additional studies are required to clarify the precise regulatory mechanisms linking ABA metabolism and ROS detoxification in heat-stressed rice anthers.

## 5. Conclusions

This study demonstrates that high-temperature stress significantly impairs reproductive success in rice, particularly affecting anther dehiscence, pollen viability, and spikelet fertility. Through a comparison of two contrasting rice genotypes, we confirmed that HTR-1 shows well-established physiological traits associated with heat tolerance, including increased antioxidant enzyme activity, reduced ROS accumulation, and a more stable phytohormonal balance. In contrast, the sensitive genotype HTS-5 showed excessive ABA accumulation, decreased GA and IAA levels, and induced oxidative damage, which contributed to pollen sterility under high-temperature stress. These findings support the role of ROS scavenging and phytohormonal regulation in adapting to high-temperature stress. Based on these findings, breeding programs can include HTR-1’s advantageous traits to improve rice’s resilience to heat stress driven by climate change. Furthermore, integrating transcriptomic and proteomic approaches in future studies could provide a deeper understanding of the regulatory pathways that improve heat tolerance in rice.

## Data Availability

The original contributions presented in this study are included in the article/supplementary material.

## Supplementary Materials

The following supporting information can be downloaded at: https://www.mdpi.com/article/10.3390/plants14050710/s1.
