# Supplementary material for: Evaluating Physiological and Hormonal Responses of Two Distinct Rice Genotypes Under High Temperatures
Source: Plants (Basel). 2025 Feb 26;14(5):710. doi: 10.3390/plants14050710 (PMC11901512; doi:10.3390/plants14050710)
Supplement: Supplementary file 1 [file plants-14-00710-s001.zip › plants-3375914-supplementary.pdf]

High temperature alters physio-biochemical markers and hormonal balance impacting anthers fertility in two contrasting rice genotypes

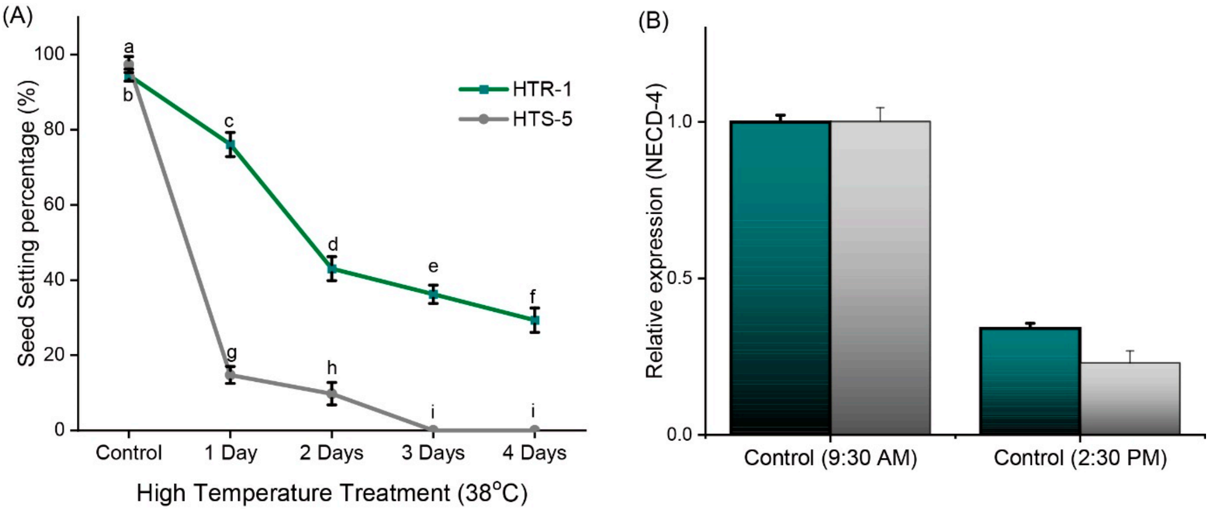

Figure S1: (A) seed setting of HTR-1 and HTS for 4 days (B) expression of OsNCED4 control conditions.

Table S1: List of primers used in the study.

qAPX1 F	TAGTCTACTACTGCTAGTAC
qAPX1 R	TAACAGCCCACCGAGACATT
qAPX2 F	AGAGTCAGTACGATCAAGAC
qAPX2 R	TCTTGACAGCAAATAGCTTGG
qAPX3 F	GCATCGATCACAATGATCG
qAPX3 R	CAGCACTCACATATATATACC
qAPX4 F	TGATGATCCAGCCATACGAACA
qAPX4 R	TTCAAAGTGCTTTTAAATAGTGAAGCT
qCATA F	CCACCACAACAACCACTACG
qCATA R	CGCGATGGGTAGTAGTCCA
qCATB F	TTCATGCACAGGGATGAAGA
qCATB R	TTCCCGACAGCCTGTTAGA
qNCED4 F	GATTGCACGGCACCTTCATT
qNCED4 R	CTCTGTAATTTGATTTTTCACTGGCTAAT
qActin1 F	TGGCATCTCTCAGCACATTCC
qActin1 R	TGCACAATGGATGGGCCAGA
